# Massive Tension Hemothorax After Pacemaker Implantation

**DOI:** 10.7759/cureus.16754

**Published:** 2021-07-30

**Authors:** Eldon Matthia, Ralph Matar, Ellery Altshuler, Richard A Kerensky, George Arnaoutakis, Samir Shah, Abdullah Omar, Zubin Agarwal, William Miles, Kun Xiang

**Affiliations:** 1 Internal Medicine, University of Florida College of Medicine, Gainesville, USA; 2 Cardiology, University of Florida College of Medicine, Gainesville, USA; 3 Thoracic and Cardiovascular Surgery, University of Florida College of Medicine, Gainesville, USA

**Keywords:** tension hemothorax, cardiac pacemaker, intercostal vessel rupture, critical hemorrhagic shock, cardiology devices

## Abstract

A case of an 85-year-old male on apixaban and clopidogrel undergoing pacemaker implantation is described. After procedure he developed unilateral tension hemothorax and required emergent drainage and exploratory thoracotomy. No vascular, cardiac, or pulmonary source was identified. After multidisciplinary discussions, it was speculated that spontaneous intercostal vessel rupture due to forceful coughing and elevated blood pressure during the procedure was the most likely cause of bleeding.

## Introduction

Hemothorax is a rare complication of pacemaker placement that has been associated with lead perforation of the right ventricle and pericardium with protrusion into the pleural space, vascular injury during venous access, or direct lung injury [1]. More rarely, hemothorax during pacemaker placement can be associated with intercostal vessel rupture with hemorrhage related to forceful cough. Rapid rise in pleural pressure has been proposed as a mechanism for rupture in at-risk patients [2]. If tension hemothorax develops, initial stabilization involves drainage of accumulated fluid, blood pressure resuscitation, and transfusion of blood products. Prompt identification and control of the hemorrhage’s source should follow. Recent instrumentation should prompt investigation of iatrogenic causes of hemothorax without discounting rare etiologies, including intercostal arterial rupture in the appropriate clinical setting. 

## Case presentation

**Figure 1:**
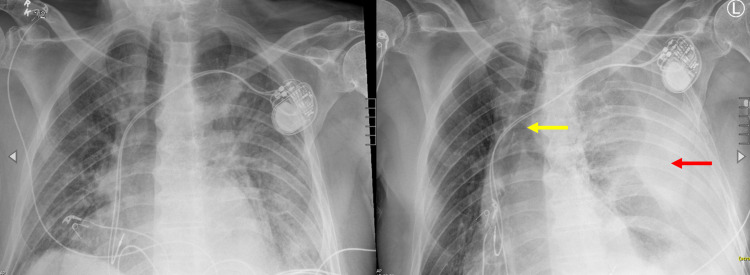
Left: Initial chest X-ray immediately after procedure. The film shows good lead position and mild, bilateral lung field infiltrates with possible small left-sided pleural effusion. Right: Emergent repeat chest X-ray showing newly appeared complete opacification of the left hemithorax (red arrow) with left lung collapse. Rightward mediastinal shift is present, as evidenced by the right-sided carina (yellow arrow).

An 85-year-old man with persistent atrial fibrillation and taking apixaban, coronary artery disease with recent coronary stenting and taking clopidogrel, hypertension, diabetes mellitus, chronic small pericardial effusion, and sick sinus syndrome was admitted for an elective dual-chamber pacemaker implantation because of symptomatic bradycardia. The procedure was performed under conscious sedation via left axillary approach. Intra-procedurally, the patient experienced frequent, forceful coughing. His blood pressure rose above 210/110 mmHg. Five milligrams intravenous metoprolol was administered. Immediate post-procedure chest X-ray showed no pneumothorax, good lead position, and possible left pleural effusion (Figure 1).

Two hours after the completion of the procedure, he became dyspneic (respiratory rate 26 breaths per minute), tachycardic (heart rate 110 beats per minute), and hypotensive (blood pressure 83/60 mmHg). Examination showed an ill-appearing patient with fluctuating mentation. His cardiopulmonary examination was significant for absent breath sounds in the left lung base.

**Figure 2:**
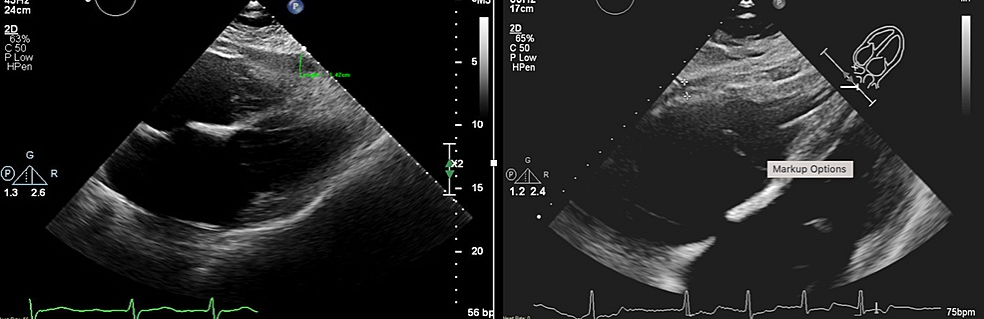
Left: Echocardiogram four months prior to procedure with chronic pericardial effusion. Right: Emergent post-procedure echocardiogram showing a new pleural effusion and a stable, small pericardial effusion without evidence of increased filling pressures.

Because of the unremarkable initial chest X-ray, a bedside transthoracic echocardiogram (TTE) was performed. It demonstrated no tamponade, a small pericardial effusion similar to that four months prior, and a pleural effusion (Figure 2). Emergent repeat chest X-ray showed complete opacification of the left hemithorax with left lung collapse and rightward mediastinal shift (Figure 1). The hemoglobin level decreased to 8.7 g/dL from baseline 12.0 g/d.

**Figure 3:**
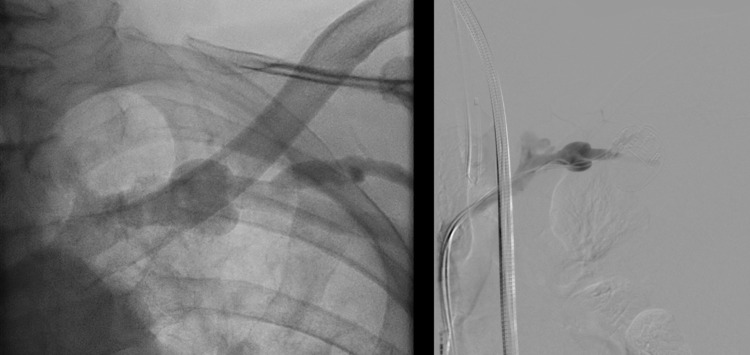
Left: Pre-procedure venogram with patency of the central vasculature. Right: Post-procedure, invasive catheter selective left subclavian venogram showed the left subclavian and axillary veins and their respective branches without evidence of contrast extravasation.

An emergent chest tube was inserted at bedside, which drained frank blood. Because the TTE showed no significant pericardial effusion, the hypothesized bleeding source was vascular injury. The patient was taken to the hybrid operating room for diagnostic evaluation. Selective left subclavian venography was performed and compared to venography immediately before pacemaker insertion (Figure 3). The left subclavian vein and branches showed no contrast extravasation.

Next, cardiac lead perforation was considered. A subxiphoid pericardial window was performed, yielding minimal serous fluid, but no blood. He remained hemodynamically unstable with ongoing blood drainage from the left chest tube. An intra-operative bronchoscopy revealed no active pulmonary bleeding, but residual blood clot was noted inside the left trachea. Therefore, the patient underwent left exploratory thoracotomy. Extensively clotted blood was evacuated from the left hemithorax. Total blood loss was estimated to be 6 L. Direct visual inspection revealed no lung parenchymal laceration. The left subclavian vein and artery and the pericardium and mediastinal surfaces remained intact. The majority of blood localized to the superior portion of the left lateral chest wall, consistent with the initial X-ray findings of latero-medial progression of the hemothorax.

**Figure 4:**
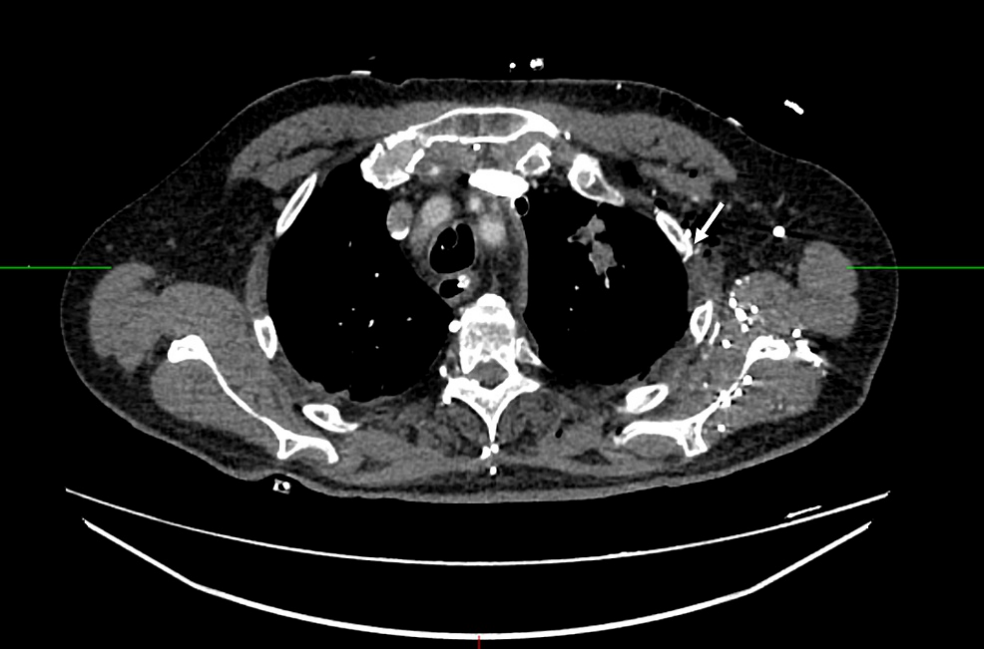
Contrast-enhanced computed tomography showed small pleural hemorrhage and third and fourth rib fractures (white arrow).

At this point the patient was hemodynamically stable with no bleeding source identified. The patient’s left thoracotomy incision was closed, and he was transferred to the cardiac intensive care unit. Computed tomography showed incidental left-lateral, non-displaced third and fourth rib fractures near the preponderance of blood, a small amount of intraparenchymal pulmonary hemorrhage near the chest tube insertion site, and no active contrast extravasation (Figure 4). The patient subsequently improved and was extubated within 24 hours. His hemoglobin remained stable after re-initiation of aspirin and apixaban. He was eventually discharged in stable condition.

## Discussion

Patients with hemothorax experience a broad range of symptoms, commonly including chest pain and dyspnea [1]. Depending on the speed and severity of the hemorrhage, they develop tachycardia, tachypnea, and hypotension related to hemorrhagic shock. Findings such as neck vein distention and tracheal deviation may indicate complications such as tamponade or tension physiology [1]. This patient developed a massive left hemothorax after ipsilateral pacemaker implantation. No clear explanation for the hemorrhage was found. Angiography and surgical visualization showed no vascular injury, a potential complication of obtaining access during pacemaker implantation. Myocardial perforation poses another possible cause, which would initially produce pericardial hemorrhage, manifesting as increased pericardial effusion [1]. Additionally, his degree of hemothorax would require considerable cardiac trauma with penetration of the myocardium and pericardium to infiltrate the pleural space. However, there was no bleeding from the pericardial window drain, and the pericardium and mediastinal surfaces appeared intact during exploratory thoracotomy. Pulmonary injury, another conceivable complication, is also unlikely given the lack of significant lung laceration during thoracotomy. Considering the preceding forceful coughing, severe blood pressure elevation during the procedure, and fractured ribs near the postulated origin of hemorrhage, the plausible bleeding source was attributed to spontaneous rupture of the left third or fourth intercostal artery, which resolved with administration of blood products.

The continuation of apixaban and clopidogrel during the procedure likely contributed to his hemorrhage. His risk of stroke was moderate to high according to his CHA2DS2-VASc score of 4, giving him an annual stroke risk of 6.7% [3]. This risk was weighed against his risk of hemorrhage prior to the procedure in accordance with American College of Cardiology (ACC)/American Heart Association guidelines. Ample evidence supports uninterrupted anti-coagulation with warfarin during pacemaker or implantable cardioverter defibrillator implantation compared to bridging therapy [4]. The evidence for a similar approach with direct oral anti-coagulants is less robust. The only randomized controlled trial to evaluate this question directly terminated early due to medical futility. The authors concluded that either approach may be reasonable, and the decision should be individualized [5]. Based on the available evidence, this procedure’s low bleeding risk, and his elevated stroke risk, it was decided to perform pacemaker implantation with uninterrupted anti-coagulation in accordance with ACC guidelines [6].

Pure hemothorax is a rare complication of pacemaker placement. Common mechanisms include pacemaker lead perforation, vascular injury, and pulmonary injury [1]. The most likely source of hemorrhage in our patient was determined to be intercostal vessel rupture, an infrequent etiology. Intercostal hemorrhage is usually traumatic, but spontaneous cases are reported, usually associated with coagulopathy, vascular fragility, neoplasm, or miscellaneous causes, including cough with or without associated rib fractures [2]. In our case, the patient’s risk was increased due to vascular fragility related to his age and uncontrolled hypertension, iatrogenic coagulopathy due to apixaban and clopidogrel, and forceful coughing.

Several cases of intercostal vessel rupture with hemorrhage related to forceful cough have been reported. Camarillo-Reyes et al. record a case of ruptured seventh intercostal artery after coughing similar to our patient’s presentation, which required embolization [7]. Ricketti et al. discuss a patient in status asthmaticus who also developed a spontaneous hemothorax requiring surgical intervention. No source could be identified, and intercostal vessel rupture was suspected [8]. Additionally, Jang and Yu both detail accounts of abdominal hematomas from ruptured intercostal arteries after forceful coughing [9,10]. Rapid rise in pleural pressure has been proposed as a mechanism for rupture in at-risk patients [9]. Treatment first requires patient stabilization with blood products and anti-coagulant reversal, if indicated [1]. If tension or tamponade physiology develops, the culprit hemorrhage must be drained immediately [1]. Embolization or surgical ligation of the causative vessel may be performed in patients who continue to hemorrhage despite conservative measures [1].

## Conclusions

Tension hemothorax without preceding trauma is a rare entity. When identified, the clinical history guides considerations of source control. Recent instrumentation should prompt investigation of iatrogenic causes without discounting rare etiologies, including intercostal arterial rupture in the appropriate clinical setting. In anti-coagulated patients at high risk of hemorrhage who undergo cardiac device implantation, the decision to continue anti-coagulation should be individualized.
